# Electrocortical Evidence for Impaired Affective Picture Processing after Long-Term Immobilization

**DOI:** 10.1038/s41598-019-52555-1

**Published:** 2019-11-12

**Authors:** Katharina Brauns, Anika Werner, Hanns-Christian Gunga, Martina A. Maggioni, David F. Dinges, Alexander Stahn

**Affiliations:** 1 Charité – Universitätsmedizin Berlin, a corporate member of Freie Universität Berlin, Humboldt-Universität zu Berlin, and Berlin Institute of Health, Institute of Physiology, Charitéplatz 1, CharitéCrossOver, Virchowweg 6, 10117 Berlin, Germany; 2 Université de Normandie, INSERM U 1075 COMETE, Caen, 14032 France; 3 Department of Biomedical Sciences for Health, Università degli Studi di Milano, via Colombo 71, 20133 Milan, Italy; 4 Department of Psychiatry, Perelman School of Medicine, University of Pennsylvania, 1016 Blockley Hall, 423 Guardian Drive, Philadelphia, PA 19004 USA

**Keywords:** Emotion, Human behaviour

## Abstract

The neurobehavioral risks associated with spaceflight are not well understood. In particular, little attention has been paid on the role of resilience, social processes and emotion regulation during long-duration spaceflight. Bed rest is a well-established spaceflight analogue that combines the adaptations associated with physical inactivity and semi-isolation and confinement. We here investigated the effects of 30 days of 6 degrees head-down tilt bed rest on affective picture processing using event-related potentials (ERP) in healthy men. Compared to a control group, bed rest participants showed significantly decreased P300 and LPP amplitudes to pleasant and unpleasant stimuli, especially in centroparietal regions, after 30 days of bed rest. Source localization revealed a bilateral lower activity in the posterior cingulate gyrus, insula and precuneus in the bed rest group in both ERP time frames for emotional, but not neutral stimuli.

## Introduction

Affective processing and emotion regulation are fundamental to human behaviour. They facilitate decision making, have significant influences on learning and memory and provide the motivation for critical action in the face of environmental incentives. The management of positive and negative emotions also directly relates to individual sociability and social interactions. Any emotional alteration may interfere with cognitive performance, impair mental well-being and lead to various forms of psychopathology, especially in the context of a stressful environment^1^. When living and working in an isolated, confined and hostile environment like deep space for prolonged durations, astronauts are exposed to numerous stressors including social isolation, confinement and weightlessness. Currently, the neurobehavioral risks associated with these stressors are not fully understood. In particular, the role of resilience, social processes and emotion regulation during long-duration spaceflight has received little attention so far. Head-down tilt bedrest (HDT) is a well-established model to simulate physical deconditioning and cephalic fluid shifts during standard space missions on the International Space Station (ISS)^2^. Bed rest also comprises a degree of sensory deprivation, isolation, and confinement^3^. Previous studies suggest that long-duration bed rest increases the risk for mood disorders^4^, and impairs emotion recognition processing during a Flanker task^5^. According to the authors’ knowledge no study has investigated the effects of long-duration bed rest on the neural correlates of emotional processing. The current study aimed to address this gap by investigating the effects of 30 days of -6 degrees HDT bed rest on cortical emotional modulation using event-related brain potentials from a standardized and well-established paradigm^6^. We hypothesized that long-term bed rest would lead to a cortical inhibition of affective processes as indicated by reduced event-related potentials.

## Results

### Emotional self-reports

Table 1 illustrates the self-reported evaluations of each picture category for the control (CTRL) group tested before bed rest, and the intervention group tested after 30 days of head-down tilt bed rest (HDBR). The ratings for all three picture categories were consistent with IAPS normative data^6^, confirming the validity of the paradigm in the present experimental setup. In both groups, positive pictures were rated as more arousing and got greater scores for valence than neutral ones (Table 1). Additionally, unpleasant slides received a lower scoring than neutral pictures for valence and were evaluated as more arousing (Table 1). This was confirmed by mixed model analyses, showing a significant main effect of stimulus condition on arousal (*F*(2,36) = 76.78, *p* < 0.001) and valence (*F*(2,36) = 309.20, *p* < 0.001).

**Table 1:** Subjective ratings for pleasant, neutral and unpleasant IAPS pictures in CTRL and HDBR group.

Group	Stimulus	Valence Rating	Arousal Rating
CTRL	pleasant	7.6 (0.6)	5.7 (1.2)
	neutral	4.6 (0.9)	2.4 (1.2)
	unpleasant	2.6 (0.5)	5.7 (1.0)
HDBR	pleasant	7.6 (0.9)	5.7 (1.6)
	neutral	5.2 (0.8)	2.2 (1.2)
	unpleasant	2.6 (0.7)	5.7 (2.2)

Note: Subjective ratings are based on 9-point Likert scales, ranging from very unpleasant/not arousing at all to very pleasant/very arousing. Data are means and standard deviations.

However, statistical analyses neither revealed a significant stimulus x group interaction, nor a significant group effect for valence (*F*(2,36) = 0.05, *p* = 0.948 and *F*(1,18) = 0.02, *p* = 0.879, respectively) or arousal (*F*(2,36) = 0.10, *p* = 0.909 and *F*(1,18) = 0.08, *p* = 0.928, respectively). Planned contrasts revealed similar ratings for valence and arousal for all picture categories between groups (all *p*s > 0.728).

### Electrophysiological data

**Figure 1:**
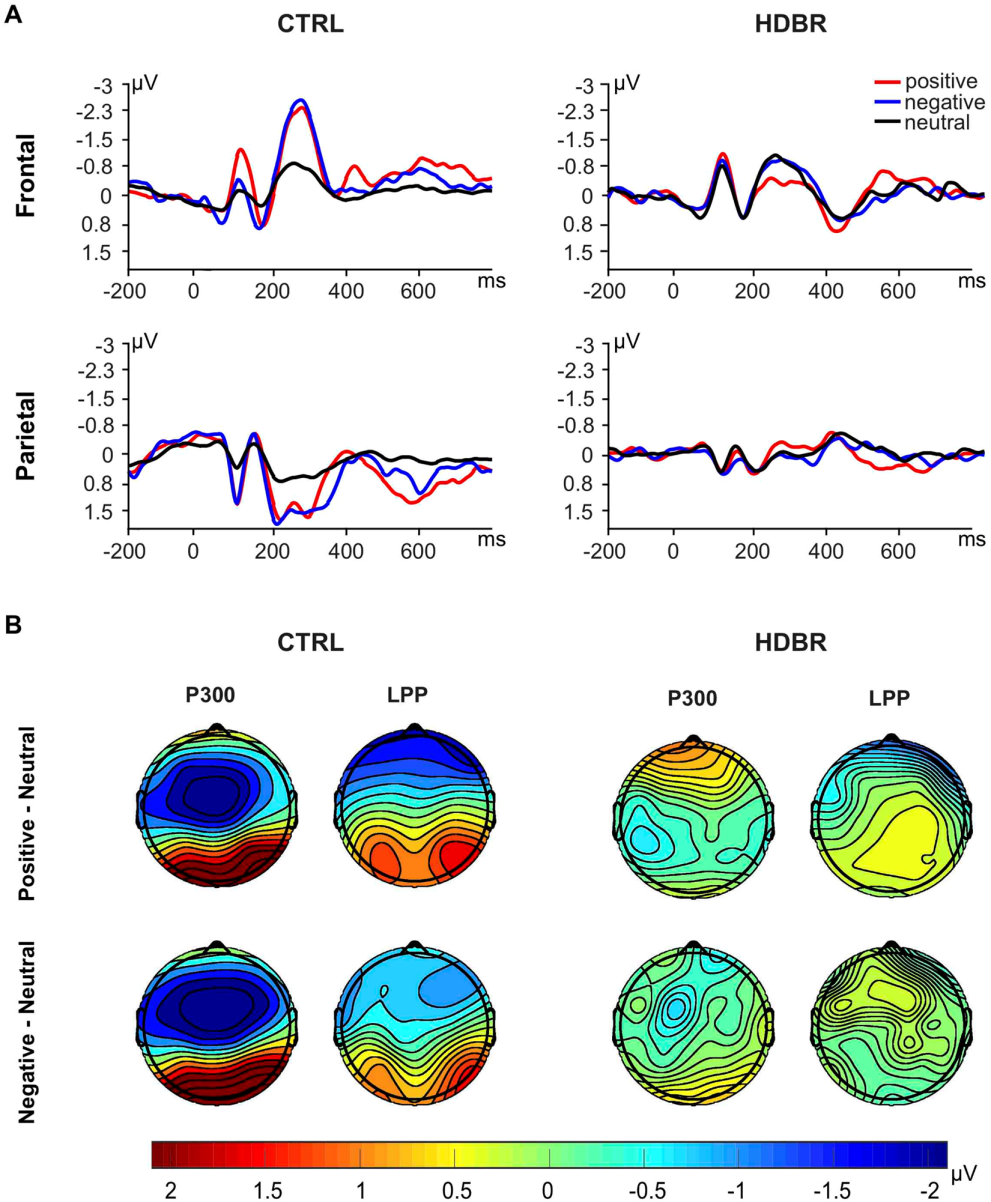
Event-related potential (ERP) results. (**A**) Grand average ERP waveforms at selected electrode clusters (frontal: F3, F4; parietal: P3, P4, Pz) for positive. (n = 25), negative (n = 25) and neutral (n = 25) stimuli in a control group (CTRL, n = 10) and a bed rest group (HDBR, n = 10). (**B**) Topographical maps depicting mean voltage differences between positive and neutral, and between negative and neutral stimuli averaged for the CTRL group and HDBR group for each ERP component (i.e., P300, and LPP).

Figure 1A depicts the grand average ERP waveforms for CTRL and HDBR subjects in frontal and parietal regions, respectively. While neutral pictures elicited similar responses in CTRL and HDBR participants, the ERP waveforms of emotional stimuli were inhibited in the HDBR group compared to the CTRL group. As shown in Table 2, the mixed ANOVA analysis of mean P300 amplitude revealed a significant interaction of group and stimulus in the frontal (*p* = 0.002) and parietal sites (*p* = 0.002). Mean LPP amplitude showed a significant effect of group in frontal (*p* = 0.048) and parietal sites (*p* = 0.026) and a significant effect of stimulus in parietal site (*p* < 0.001). Simple comparisons are shown in Table S1 and Table S2 that can be found in the Supplementary Information.

**Table 2:** Mixed-model analyses assessing the effects of group (HDBR, CTRL) and stimuli (negative, postive, neutral) on P300 and LLP components.

Factor	P300	LLP
Frontal electrode cluster		
Group	*F*(1, 18) = 2.84	*F*(1, 18) = 4.50*
Stimulus	*F*(2, 36) = 10.12***	*F*(2, 36) = 1.33
Group x Stimulus	*F*(2, 36) = 7.22**	*F*(2, 36) = 3.03
Parietal electrode cluster		
Group	*F*(1, 18) = 11.16**	*F*(1, 18) = 5.86*
Stimulus	*F*(2, 3 6) = 3.72*	*F*(2, 36) = 16.48***
Group x Stimulus	*F*(2, 36) = 7.65**	*F*(2, 36) = 2.21

*p < 0.05. **p < 0.01. ***p < 0.0001.

Planned contrasts (Table S1) confirmed that emotional pictures induced enhanced electrocortical responses in CTRL compared to HDBR participants in both regions and time frames (all *p*s < 0.029) except for the frontal LPP which was not significant between groups for positive pictures (*p* = 0.074). For the neutral stimuli, no differences in LPP and P300 amplitudes between groups were observed (all *p*s > 0.314). The ERP difference topography between emotional and neutral stimuli for both components and both groups is illustrated in Fig. 1B. While CTRL participants showed enhanced P300 and LPP amplitudes for emotional stimuli relative to neutral pictures, there was no visible difference in the HDBR group. A follow-up analysis using pre-planned contrasts (Table S2) revealed that positive and negative stimuli evoked significantly increased P300 components compared to neutral stimuli in the CTRL (all *p*s < 0.003), but not the HDBR group (all *p*s > 0.414). We also observed significant differences between LPP components induced by positive stimuli and neutral stimuli in both regions (all *p*s < 0.037) and a significantly smaller LPP amplitude in the frontal area induced by negative pictures compared to neutral pictures (*p* < 0.001) in CTRL participants only.

### eLORETA data

**Figure 2:**
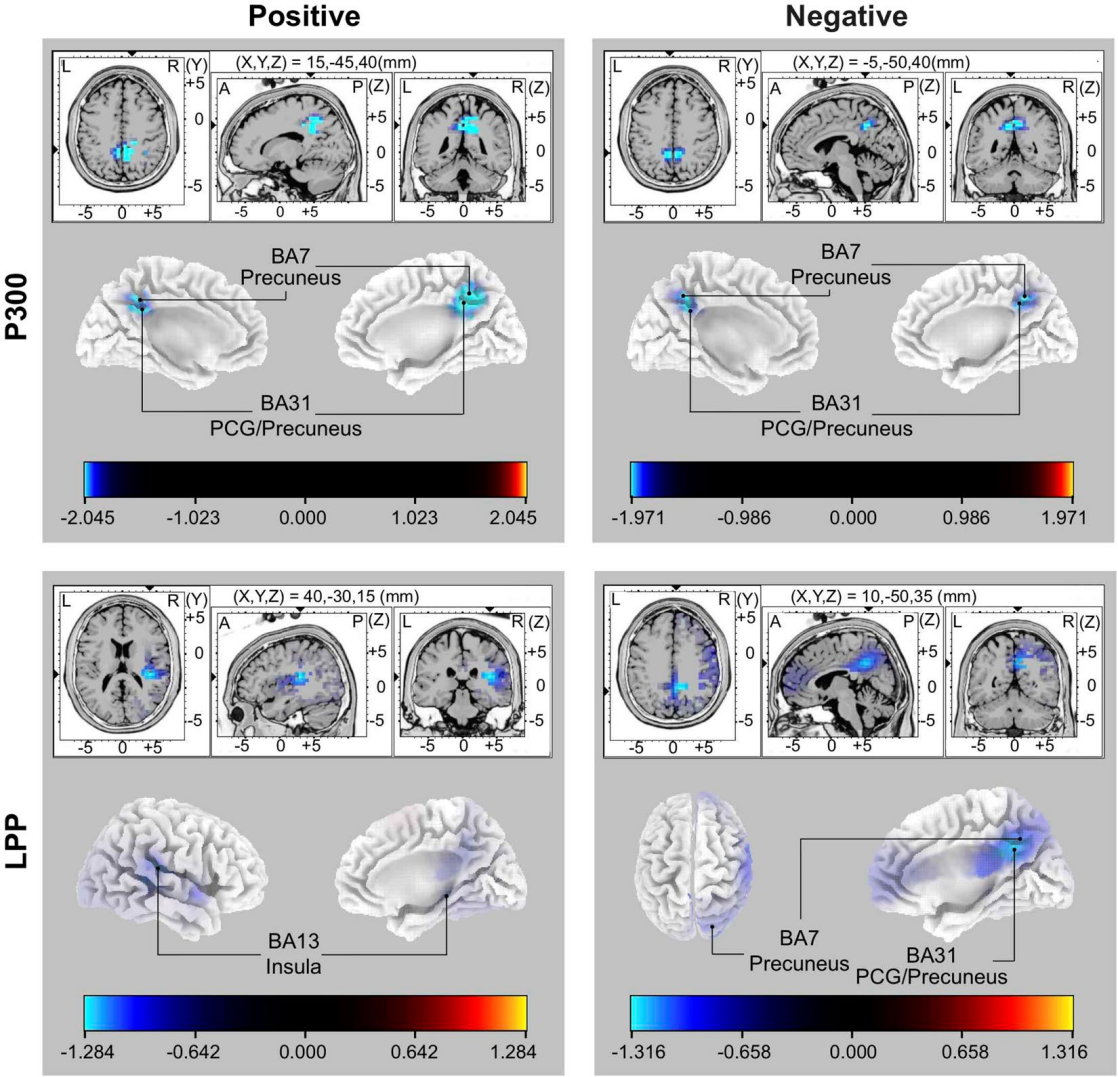
Statistical parametric maps (SPMs) indicating the differences in brain source localization between control (CTRL, n = 10) and head-down-tiltbed rest group (HDBR, n = 10). Data for positive and negative stimuli are shown on the left and right panels, respectively. Results for the P300 and LPP components are provided in the upper and lower panels, respectively. Blue colours indicate decreased activity in the HDBR compared to the CTRL group. The color scale indicates F-values for group differences of brain activity. L left, R right, A anterior, P posterior, PCG posterior cingulate gyrus, BA Brodmann area.

For the averaged LPP evoked by positive pictures, a significantly lower cortical activation for HDBR compared to CTRL participants was found in the right insula (BA 13, *p* < 0.05, Fig. 2). The P300 comparison between CTRL and HDBR group revealed statistically lower cortical activations in the bilateral precuneus and the bilateral cingulate gyrus (BA 31/7, *p* < 0.05, Fig. 2). Moreover, analysis of P300 and LPP showed a decrease in cortical activity at the same locations (BA 31/7; all *p*s < 0.05, Fig. 2) when processing negative pictures, as compared to CTRL group. No significant differences were found comparing CTRL and HDBR group for mean P300 and LPP amplitudes evoked by neutral stimuli (see *F*_critical_ in Table 3).

**Table 3:** Loreta critical thresholds (*F*_critical_) and maximal *F*-statistics (*F*_max_) for ERP components and each stimulus type.

		Positive	Neutral	Negative
P300	*F*_critical_ for *p* < 0.05	1.85	1.71	1.78
	*F*_critical_ for *p* < 0.01	2.05	1.91	1.97
	Statistical Threshold	−2.48	−1.53	−2.02
LPP	*F*_critical_ for *p* < 0.05	1.24	1.31	1.23
	*F*_critical_ for *p* < 0.01	1.37	1.51	1.39
	*F*_max_	−1.28	−0.88	−1.32

## Discussion

The present study investigated the effects of 30 days of immobilization on affective picture processing in young healthy men. To evaluate the impact of long-term bed rest on emotional processing we employed a well-established ERP paradigm using standardized affective stimuli. Our main findings include an inhibition of P300 and LPP components for emotional stimuli, but not neutral pictures in HDBR participants when compared to a sex- and age-matched control group. This inhibition was found to be localized in the precuneus, cingulate gyrus, and insula.

The CTRL group exhibited larger P300 and LPP components when viewing pleasant and unpleasant pictures as compared to neutral slides. This result is well in line with previous research investigating affective picture processing in young healthy adults^7,8^. Larger evoked potentials are thought to reflect increased attention towards biologically relevant emotional stimuli^9^. Particularly, the P300 has been hypothesized to be an index of initial memory storage and attention^10^, whereas the LPP is supposed to be a cortical correlate that is associated with encoding and memory processes^11^. Additionally, emotional stimuli are better perceived, encoded, consolidated and retrieved than neutral stimuli^12^. In contrast, we did not observe the expected difference between brain potentials in HDBR participants, immobilized for 30 days in -6 degrees head-down tilt position. We found that long-term immobilization resulted in emotional blunting as evidenced by reduced LPP and P300 amplitudes in response to affective images, i.e., pleasant and unpleasant stimuli elicited a similar flattened response as neutral ones. The emotional blunting indicates dysfunctional modulations in the processing of emotional information.

A source localization revealed a cortical inhibition of distinct brain regions. Specifically, long-term bed rest was found to be associated with a lower activation within the right insula, the bilateral precuneus, and the bilateral posterior cingulate gyrus (PCG) when processing pleasant and unpleasant stimuli. Electrophysiological recordings and neuroimaging have supported key positions of the amygdala, cingulate gyrus and insula in response to emotional stimuli^13^. Moreover, past studies reported a similar role in emotional information processing for PCG and precuneus due to their structural and functional similarities^14^. There is current evidence, that PCG and precuneus are activated during the evaluation of emotional words^15^, the retrieval of emotional memories^16^ and the processing of self-relevant affect^17^. The insula, however, plays an important role in pain processing^18^ and, additionally, has been shown to be instrumental in the detection, interpretation, and regulation of internal bodily states^19^, therefore serving as a critical bridge between affective and cognitive processes. Moreover, precuneus, PCG and insula are reciprocally connected to areas involved in emotional processing such as the anterior cingulate and the orbital frontal cortices, as well as the amygdala^20,21^. Considering these findings, it is reasonable that PCG, precuneus and insula carry emotion-specific information. Interestingly, Zou and colleagues recently showed that 45 days of bed rest altered the resting-state functional architecture of a similar region including the insula and cingulate cortex and hypothesized that these effects might influence the processing of salient information^22^. These data support the vulnerability of these structures to the detrimental neurocognitive effects of prolonged immobilization.

Notably, the self-evaluation of valence and arousal did not differentiate our two groups. The absence of any differences indicates that physiological data may be more objective than behavioural measures as they do not underlie cognitive-social control and are therefore less sensitive to experimental manipulations. Participants possibly tend to respond to self-evaluation in a stereotyped fashion. In line with this, Messerotti and colleagues have shown that acute HDT can suppress cortical emotional responses^23^, without affecting behavioural responses. They attribute the electrocortical changes to an altered body position. Recent research performed by the same group has demonstrated that these postural effects on electrocortical activity are immediately observed after changing from sitting to the supine position^24^. To account for postural effects in the present experiment, both groups were tested in the same position, i.e., at -6 degrees HDT, providing sufficient time to account for the cephalic fluid shifts^25^. We therefore assume that the present findings are explained by mechanisms other than acute postural effects.

HDT leads to alterations in brain hemodynamics including an increase in cerebral blood flow (CBF), intracranial pressure, and oxygenated haemoglobin^26^, which are hypothesized to trigger cortical inhibition^27^. Additionally, HDT is associated with a cephalic fluid shift leading to increases in thoracic blood volume and hydrostatic pressure, stimulating cardiopulmonary and arterial baroreceptors^2^. These cardiovascular dynamics have been shown to affect cortical activation. Arterial baroreceptors can inhibit cortical activity^28^ by decreasing locus coeruleus activity and cortical noradrenaline turnover^29^. Likewise, the blunted responses in HDBR subjects might also be explained by neuroendocrine changes associated with bed rest. Several neurotransmitters are known to be decreased by inactivity including serotonin and norepinephrine^30^. The monoaminergic system which includes norepinephrine and serotonin is well-known for its critical role in controlling human behaviour^31^ and in several psychiatric disorders such as depression^32^, anxiety^33^, and behavioural disturbances among people with dementia^34^. A change in monoamine concentrations associated with long-duration immobilization^35^ could therefore also contribute to the changes in visual affective processing observed in the present study. Future studies should therefore also combine behavioural, brain functional, cardiovascular and neuroendocrine measures that will allow to better understand such mechanisms. We also acknowledge that we chose a between-subjects design to exclude any learning effects. Direct between-subject comparisons can be biased by various factors associated with the heterogeneity of the two groups. However, all participants underwent intensive psychological and medical screening for their inclusion in the bed rest study, and they were carefully matched and randomly assigned to one of the two groups. Resting state EEG measured eight days before the intervention, confirmed that EEG spectral power did not differ between the two groups. However, future studies are certainly needed to verify these findings using a within-subjects design in a larger cohort.

Taken together, our data show that head-down tilt bed rest can have adverse neurobehavioral effects associated with negative and positive valence. Impaired affective picture processing following prolonged bed rest was evidenced by a reduction in LPP and P300 in specific brain areas including the insula, precuneus and cingulate gyrus. These results highlight the pervasive effects of physical inactivity that go beyond cardiovascular and musculoskeletal deconditioning. They could have important implications for situations, in which physical activity levels are markedly limited such as during long-duration spaceflight, the aging population, in bed-confinement during hospitalized based care, and people with sedentary lifestyles. Future research needs to elucidate the mechanisms underlying the effects of physical inactivity, examine inter- and intraindividual vulnerabilities relative to emotional regulation, and identify the interaction of physical inactivity and other stressors.

## Methods

The present experiment was part of a European Space Agency (ESA) sponsored bed rest study performed at the facilities of the French Institute for Space Medicine and Physiology (MEDES), Toulouse, France in 2017. The project has been registered in the Clinical Trial.gov database under NCT03594799. It comprised 15 days of baseline data collection, 60 days of -6 degrees HDT bed rest and 15 days of recovery. It was conducted following the Declaration of Helsinki for Medical Research Involving Human Subjects and approved by the Comité de Protection des Personnes (CPP Sud-Ouest Outre-Mer I), the French Health Authorities (Agence Française de Sécurité Sanitaire des Produits de Santé) and the Ethics Committee at Charité–Universitätsmedizin Berlin. All participants were informed about the purpose, experimental procedures, and risks before giving their verbal and written informed consent.

### Participants

Data was collected from 20 young healthy male participants (mean age = 34 years, SD = 8; mean height = 176 cm, SD = 4.7; mean weight = 74.0 kg, SD = 7.1; n = 17 right-handed). Handedness was assessed using the Edinburgh Handedness Inventory^36^. Sample sizes were based on previous bed rest studies, suggesting neurobehavioral effects for bed rest^4,5^. We also performed sensitivity analyses for our main outcome, i.e., the comparison of ERP between the bed rest (HDBR) and the control (CTRL) group. For a two-sided independent t-test, a level of significance of 0.05, and a power of 80%, a significant difference corresponding to a Cohen’s d of 1.32 should be detectable. This effect is much larger than in a previously reported study using the identical paradigm to assess the acute effects of head-down tilt bed rest^23^. We were therefore confident that the current sample size would be sufficient to reveal a significant between-subjects effect for our primary outcome. All volunteers had no personal history of neurological or psychiatric illness, drug or alcohol abuse, or current medication, and they had a normal or corrected-to-normal vision. The subjects were randomly assigned to one of two groups in a counterbalanced fashion. One of the group served as a control (CTRL: mean age = 34 years, SD = 7; mean height = 176 cm, SD = 3.5; mean weight = 73.1 kg, SD = 5.4) and was tested 8 days prior to bed rest in a -6 degrees HDT position after an adaptational period of 30 minutes of rest. The experimental group (HDBR: mean age: 34 years, SD = 8; mean height = 176 cm, SD = 5.6; mean weight = 74.9 kg, SD = 6.5) was tested after 30 days of (-6 degrees HDT) immobilization. Study cohorts did not differ in age and anthropometric factors (all *p*s > 0.740). Moreover, spectral power analysis of resting state EEG data collected eight days before bed rest revealed no significant difference between groups (data not shown, *p* = 0.420).

### Stimuli

Seventy-five standardized stimuli were selected from the IAPS dataset^6^ including unpleasant (n = 25, e.g., scenes of violence, threat and injuries), pleasant (n = 25, e.g., sporting events, erotic scenes) and neutral pictures (n = 25, e.g., household objects, landscapes) and presented in a random order. The normative valence ratings (mean (SD)) for each picture category were 7.55 (0.40), 4.99 (0.26), and 3.00 (0.81), and the normative arousal levels (mean (SD)) for each stimulus type were 6.31 (1.10), 2.63 (0.52) and 5.19 (0.61) for positive, neutral and negative images, respectively. The catalogue numbers of pictures from the IAPS dataset used in this study can be found in Supplementary Information.

### Procedure

Subjects were positioned in -6 degrees HDT in a dimly lit sound-attenuated room. Testing was performed using a desktop computer (PCGH-Supreme-PC, Alternate), with a 21.5-in monitor (Iiyama ProLite, 1 ms response time, 55–75 Hz refresh rate, luminance 250 cd/m2) installed approximately 60 cm apart from the participant. Before each trial, a central fixation cross appeared for 500 ms. Pictures were displayed on the screen for 2000 ms. After each picture presentation participants were asked to rate the arousal and valence of their emotional perception using two independent 9-point self-assessment Likert scales (SAM) that ranged from very unpleasant/not arousing at all to very pleasant/very arousing^37^. The rating was performed using a computer mouse without any time constraints. The accuracy was emphasized to ensure response reliability and maximal attention from the subjects to their feelings.

### EEG recording

The electrocortical activity was continuously recorded and synchronized with the stimuli using an active electrode 32-channel amplifier (actiCHamp, Brain Products GmbH, Germany). Picture presentation and timing were controlled through the use of Presentation software version 18.1 (Neurobehavioral Systems, Inc., USA). Electrodes were attached to an EEG cap (actiCap, Brain Products GmbH, Germany) and placed at positions Fp1, F3, FT9, FC5, FC1, T7, TP9, CP5, CP1, P7, P8, TP10, CP6, CP2, T8, FT10, FC6, FC2, Fp2, F7, F8, F3, F4, Fz, C3, C4, Cz, P3, P4, Pz, O1 and O2 in accordance with the International 10–20 System. Signals were referenced to Fz. Electrode impedance was checked for each subject before data collection and maintained at less than 5 kΩ. Eye movements and eye blinks were monitored via tin electrooculogram (EOG) electrodes (B18 Multitrodes, EASYCAP GmbH, Germany) placed above and below the left eye as well as at the outer canthi of both eyes. EEG and EOG signals were amplified by a multi-channel bio-signal amplifier and A/D converted at 1000 Hz per channel with 24-bit resolution.

### EEG data processing

The data were analysed offline employing EEGLAB 14.0.0^38^, a toolbox embedded in Matlab R2015b (The MathWorks, Inc., Natick, Massachusetts, United States). First, data were filtered using a 0.1 to 40 Hz band pass filter. Then, recordings were visually inspected allowing also an interpolation of bad channels. After re-referencing to average reference, EEG data were epoched to the respective stimulus presentation including 200 ms of pre-stimulus baseline and 800 ms of stimulus-dependent data. EOG artefacts were removed using vertical and horizontal EOG regression channels^39^. Muscle artefacts were removed using a spatial filtering framework with defaults^40^. After baseline removal, ERPlab 6.1.3^41^ was used to run an additional automated exclusion procedure, rejecting epochs which exceed a gradient threshold of 50 μV, or a maximum and minimum amplitude of ± 100 μV. A total of 2.1% of the trials were excluded in the CTRL group, while 1.1% of the trials had to be excluded for the HDBR group. Average ERPs were computed separately for each subject and each condition. Further, the waveforms were transformed into topographic maps of the ERP potential distributions. The LPP was measured as the average voltage of 400 to 700 ms following picture onset. The P300 was measured as the average voltage of 280 to 350 ms after stimulus presentation. Mean P300 and LPP amplitude was averaged for F3 and F4 as well as P3, P4 and Pz to assess frontal and parietal activity, respectively. A digital 12 Hz low-pass filter was applied offline for plotting grand-averaged waveforms while electrophysiological activity using original filter settings was used for all statistical analyses.

### Time-dependent cortical localization of EEG activity

Source analysis was performed by exact low-resolution brain electromagnetic tomography (eLORETA, http://www.uzh.ch/keyinst/loreta.htm), enabling the spatial identification of the cortical activity. The eLORETA software employs a discrete, three-dimensional distributed, linear, weighted minimum norm inverse solution method. The particular weights used in eLORETA allow for an exact localization to test point sources and provide better localization of highly correlated point sources with low signal to noise ratio data^42^. Three-dimensional solution space is restricted to cortical gray matter, as determined by the probabilistic Talairach Atlas. The brain compartment includes 6239 voxels with 5 mm spatial resolution. Anatomical labels, i.e., Brodmann areas (BA) are reported using MNI space, with correction to Talairach space.

In order to receive the 3D cortical distribution of the electrical neuronal generators, the electrode positions were applied to a probabilistic anatomical template of the Talairach Atlas. The Talairach coordinates were used to compute the eLORETA transformation matrix. The eLORETA files were obtained, using the transformation matrix and the ERP data of each subject for each stimuli type. The transformed eLORETA files, containing the corresponding 3D cortical distribution of the electrical neuronal generators, were used for further statistical analysis.

### Statistical analysis

#### Differences in the temporal dynamics of ERP maps

Descriptive statistics are reported as means and standard deviations (SD). To test for differences in self-reported evaluations of emotional valence and arousal we performed two-factorial mixed linear models. Subjects were entered as random factors and group (CTRL, HDBR) and stimulus type (positive, neutral, negative) were included as fixed factors, respectively. Further, a mixed-model design was employed to compare the ERP components between groups (CTRL, HDBR) and stimulus type (positive, negative, neutral). Separate mixed model ANOVAs were run for each combination of region (frontal, parietal) and ERP component (P300, LPP). Stimulus type and group were entered as fixed factors and subjects as random effects. Simple comparisons for each condition were performed using pre-planned contrasts with corrections for multiple comparisons^43^. Effect sizes were reported as Cohen,s *d*. Confidence intervals of effect sizes were bootstrapped using 2000 resamples^44^. All statistical analyses were carried out using the software package R version 3.5.1^45^. Mixed models were run using the packages lme4^46^ and lmerTest2^47^. The level of significance was set at α = 0.05 (two-sided) for all testing.

#### Time-dependent localization of significant differences in temporal dynamics

Independent sampled F-tests were used to test for differences in estimated cortical current density between CTRL and HDBR in all emotional conditions and both time frames. Statistical significance was assessed using a non-parametric randomization test with 5000 randomizations that determined the critical probability threshold (F_critical_) with corrections for multiple testing^48^. As a result, each voxel was assigned a F-value. Voxel-by-voxel F-values are displayed as statistical parametric maps (SPMs).

## Supplementary information

Supplementary Information

## Data Availability

The datasets that support the findings of the current study are available from the corresponding author on reasonable request.
