# Supplementary material for: Electrocortical Evidence for Impaired Affective Picture Processing after Long-Term Immobilization
Source: Sci Rep. 2019 Nov 12;9:16610. doi: 10.1038/s41598-019-52555-1 (PMC6851182; doi:10.1038/s41598-019-52555-1)
Supplement: Supplementary file 1 — Supplementary Information [file 41598_2019_52555_MOESM1_ESM.pdf]

Electrocortical Evidence for Impaired Affective Picture Processing after Long-Term Immobilization

Katharina Brauns, Anika Werner, Hanns-Christian Gunga, Martina Maggioni, David F. Dinges, Alexander Stahn

Supplementary Information

Supplementary Study Materials

Study stimuli

The following pictures from the International Affective Picture System, listed by catalog number, were used in the current study: negative - 1114, 1205, 1110, 3064, 3140, 3168, 3250, 3261, 9140, 9265, 9301, 9419, 9420, 9433, 9520, 9592, 9800, 9925, 9926, 9561, 9341, 9342, 9340, 3230, 9584; neutral - 2235, 7057, 5390, 5731, 5740, 7002, 7004, 7006, 7009, 7010, 7056, 7025, 7041, 7050, 7052, 7053, 7055, 7059, 7060, 7080, 7090, 7150, 7175, 7233, 7235; positive - 1440, 8185, 1460, 5629, 1710, 1722, 1750, 2071, 8260, 2311, 8185, 4002, 4006, 4141, 4142, 4180, 4225, 4232, 4250, 4255, 4652, 4659, 4694, 4695.

Supplemental Results

Supplemental Study Table

Table S1. Contrasts comparing ERPs for negative, neutral and positive stimuli between control (CTRL) and bed rest (HDBR) groups.[*]

ERP	Stimulus	*df*	*t*	*p*	*d* [95%CI]
P300 frontal	negative	30	2.52	0.017	-1.15 [-1.98, -0.31]
	neutral	30	-0.45	0.659	0.30 [-0.65, 1.37]
	positive	30	2.29	0.029	-0.81[-1.70, -0.06]
P300 parietal	negative	30	-3.81	<0.001	1.56 [0.69, 2.22]
	neutral	30	-1.03	0.314	0.78 [-0.11, 1.63]
	positive	30	-3.93	<0.001	1.45 [0.75, 2.04]
LPP frontal	negative	39	2.90	0.006	-1.25 [-1.09, 0.73]
	neutral	39	0.33	0.745	-0.25 [-0.65, 1.37]
	positive	39	1.83	0.074	-0.65 [-1.69, 0.35]
LPP parietal	negative	44	-2.37	0.022	0.98 [0.13, 1.96]
	neutral	44	-0.44	0.659	0.21 [-0.83, 1.01]
	positive	44	-2.65	0.011	1.21 [-0.10, 2.19]

[*]*df*, degrees of freedom; *d*, effect size (Cohen's *d*) and 95% confidence intervals (CI). CIs are bootstrapped using 2000 resamples.

Supplemental Study Table

Table S2. Contrasts comparing ERPs between stimuli conditions (negative vs. neutral and positive vs. neutral) in control (CTRL) and bed rest (HDBR) groups.*

ERP	Group	Stimulus	*df*	*t*	*p*	*d* [95% CI]
P300 frontal	CTRL	negative - neutral	36	-5.52	<0.001	-1.76 [-2.76, -1.02]
		positive - neutral	36	3.17	0.003	-0.71 [-1.39, -0.06]
	HDBR	negative - neutral	36	-0.69	0.497	-0.21 [-0.86, 0.54]
		positive - neutral	36	1.29	0.414	0.48 [-0.35, 1.26]
P300 parietal	CTRL	negative - neutral	36	4.25	<0.001	1.19 [0.58, 1.75]
		positive - neutral	36	3.62	<0.001	0.82 [0.19, 1.43]
	HDBR	negative - neutral	36	-0.43	0.669	-0.12 [-0.82, 0.56]
		positive - neutral	36	-1.27	0.424	-0.48 [-1.17, 0.18]
LPP frontal	CTRL	negative - neutral	36	-2.36	0.037	-0.88 [-1.53, -0.19]
		positive - neutral	36	-2.17	0.037	-0.65 [-1.48, -0.06]
	HDBR	negative - neutral	36	1.11	0.552	0.45 [-0.26, 1.36]
		positive - neutral	36	-0.14	0.891	-0.04 [-0.74, 0.69]
LPP parietal	CTRL	negative - neutral	36	1.22	0.230	0.42 [-0.26, 1.23]
		positive - neutral	36	4.89	<0.001	1.88 [0.62, 3.98]
	HDBR	negative - neutral	36	-1.16	0.252	-0.30 [0.62, 3.98]
		positive - neutral	36	2.17	0.074	0.57 [-0.02, 1.15]

**df*, degrees of freedom; *d*, effect size (Cohen's *d*) and 95% confidence intervals (CI). CIs are bootstrapped using 2000 resamples.
